# Immunological Landscape of Retinal Ischemia-Reperfusion Injury: Insights into Resident and Peripheral Immune Cell Responses

**DOI:** 10.14336/AD.2024.0129

**Published:** 2024-01-29

**Authors:** Shan He, Cuiying Liu, Changhong Ren, Heng Zhao, Xuxiang Zhang

**Affiliations:** ^1^Department of Ophthalmology, Xuanwu Hospital, Capital Medical University, Beijing, China.; ^2^School of Nursing, Capital Medical University, Beijing, China.; ^3^Institute of Hypoxia Medicine, Xuanwu Hospital, Capital Medical University. Beijing, China.; ^4^Beijing Institute of Brain Disorders, Capital Medical University, Beijing, China.

**Keywords:** Retinal ischemia-reperfusion injury, immune cell, inflammation, inflammasome, retinal ganglion cell

## Abstract

Retinal ischemia-reperfusion injury (RIRI) is a complex condition characterized by immune cell-mediated inflammation and consequent neuronal damage. This review delves into the immune response mechanisms in RIRI, particularly emphasizing the roles played by resident and peripheral immune cells. It highlights the pivotal role of microglia, the primary resident immune cells, in exacerbating neuroinflammation and neuronal damage through their activation and subsequent release of pro-inflammatory mediators. Additionally, the review explores the contributions of other glial cell types, such as astrocytes and Müller cells, in modulating the immune response within the retinal environment. The dual role of the complement system in RIRI is also examined, revealing its complex functions in both safeguarding and impairing retinal health. Inflammasomes, triggered by various danger signals, are discussed as crucial contributors to the inflammatory pathways in RIRI, with an emphasis on the involvement of different NOD-like receptor family proteins. The review further analyzes the infiltration and impact of peripheral immune cells like neutrophils, macrophages, and T cells, which migrate to the retina following ischemic injury. Critical to this discussion is the interplay between resident and peripheral immune cells and its implications for RIRI pathophysiology. Finally, the review outlines future research directions, focusing on basic research and the potential for clinical translation to enhance understanding and treatment of RIRI.

## 1. Introduction

Retinal ischemia-reperfusion injury (RIRI) is a complex pathophysiological event that occurs when the retina's blood supply is cut off and then restored. This sudden change often leads to the irreversible death of retinal ganglion cells (RGCs) and subsequent damage to retinal tissue [1]. Retinal ischemia can be precipitated by a myriad of factors, including vessel occlusion, as seen in central retinal artery occlusion, chronic ischemia, hypoxia as in diabetic retinopathy, and elevated intraocular pressure (IOP) typically present in acute glaucoma. Following these ischemic events, blood flow is restored, a phase known as reperfusion.

The retina, requiring the highest metabolic demand of any tissue in the body, is serviced by an extensive dual blood supply from the choriocapillaris and the central retinal artery [2]. Consequently, its susceptibility to ischemic injury is pronounced. Even transient retinal ischemic episodes can culminate in permanent tissue damage and irrevocable vision loss, gravely impacting a patient's quality of life. Despite current therapeutic strategies, the prognosis for patients experiencing RIRI remains dishearteningly suboptimal. The onset of retinal ischemia induces cell death, predominantly necrosis, with the subsequent reperfusion sparking a deleterious cascade of events [3]. These include oxidative stress [4], glutamate excitotoxicity [3], nitric oxide release [5, 6], retinal acidosis, and intracellular calcium homeostasis disturbance [7], all of which propagate further damage and inflammation within the tissue. Significantly, ischemia induces neuronal death alongside glial activation, which results in the release of cytokines and the attraction of peripheral immune cells [8, 9]. These cells are drawn from the bloodstream to the injury site via the compromised blood-retinal barrier (BRB), inciting an inflammatory response. The inflammatory response in RIRI is primarily orchestrated by peripheral immune cells and resident immune cells within the retina, which are delivered to the injury site during the reperfusion phase. The research realm delving into this intricate mechanism is rich but multifaceted, suggesting numerous consequences arising from RIRI. Nonetheless, current research trajectories seem somewhat scattered and fail to offer a holistic perspective encompassing the phenomenon.

Notably, one aspect under-reviewed in the existing literature is the interaction between peripheral immune cells and resident immune cells in the retina. Understanding this dynamic is crucial, as these interactions are integral to the inflammatory response in RIRI and could potentially unveil new therapeutic targets. Therefore, in this review, we aim to bridge this gap in the literature by focusing on the role of immune cells in the inflammatory response in RIRI. Specifically, it investigates the immune privilege of the retina, the animal models of RIRI, the different cell types implicated in RIRI and their respective roles, the contribution of the complement system in RIRI, and the activation of inflammasomes in the inflammatory response. By synthesizing insights from various pertinent studies, we aim to offer a more unified understanding of the interaction between peripheral immune cells and native retinal cells in RIRI, an aspect previously under-investigated.

## 2. In vivo and in vitro RIRI models

Our comprehension of RIRI has significantly advanced due to the employment of both in vivo and in vitro systems replicating the disease's intricacies. The two primary animal prototypes employed for this aim are the raised IOP-induced model [10] and the middle cerebral artery occlusion (MCAO) model [11].

The elevated IOP-induced model is incredibly potent for replicating RIRI. The technique involves a temporary increase in IOP surpassing systolic levels. This pressure rise is then promptly returned to normal, effectively triggering an ischemia-reperfusion episode. This system not only aids in deciphering the sophisticated dynamics of RIRI but also furnishes essential knowledge about the interplay between neural and vascular injuries — a pivotal element in the onset of glaucoma and diabetic retinopathy [12]. Notably, the duration of pressure elevation and its magnitude can be modulated to provoke different levels of ischemia, facilitating the exploration of varied pathological scenarios.

Conversely, the MCAO model involves placing an intra-luminal filament within the middle cerebral artery, which is later extracted to initiate ischemia and reperfusion in the retina [11]. This system is frequently employed due to its capacity to mimic a physiologically pertinent scenario and illustrate the effects of short-lived ischemic episodes on retinal structures. This model can yield essential insights into the pathophysiological processes activated by RIRI and possibly reveal therapeutic avenues [13].

For in vitro examinations, the oxygen-glucose deprivation reperfusion (OGDR) system has become increasingly popular [14]. It's crafted to simulate the setting of retinal neurons and glial cells during brief ischemic and oxygen-deprived states. This framework is particularly beneficial in breaking down cellular and molecular trajectories since it provides a regulated milieu for initiating ischemia and reperfusion. The OGDR model thus paves the way for an exhaustive analysis of molecular shifts and the foundational processes of RIRI [14].

In essence, these models each provide unique and complementary avenues to deepen our understanding of the mechanisms driving RIRI and develop effective therapeutic interventions.

## 3. Retina is an immune-privileged site, and overall immune responses in RIRI

**Figure 1.**
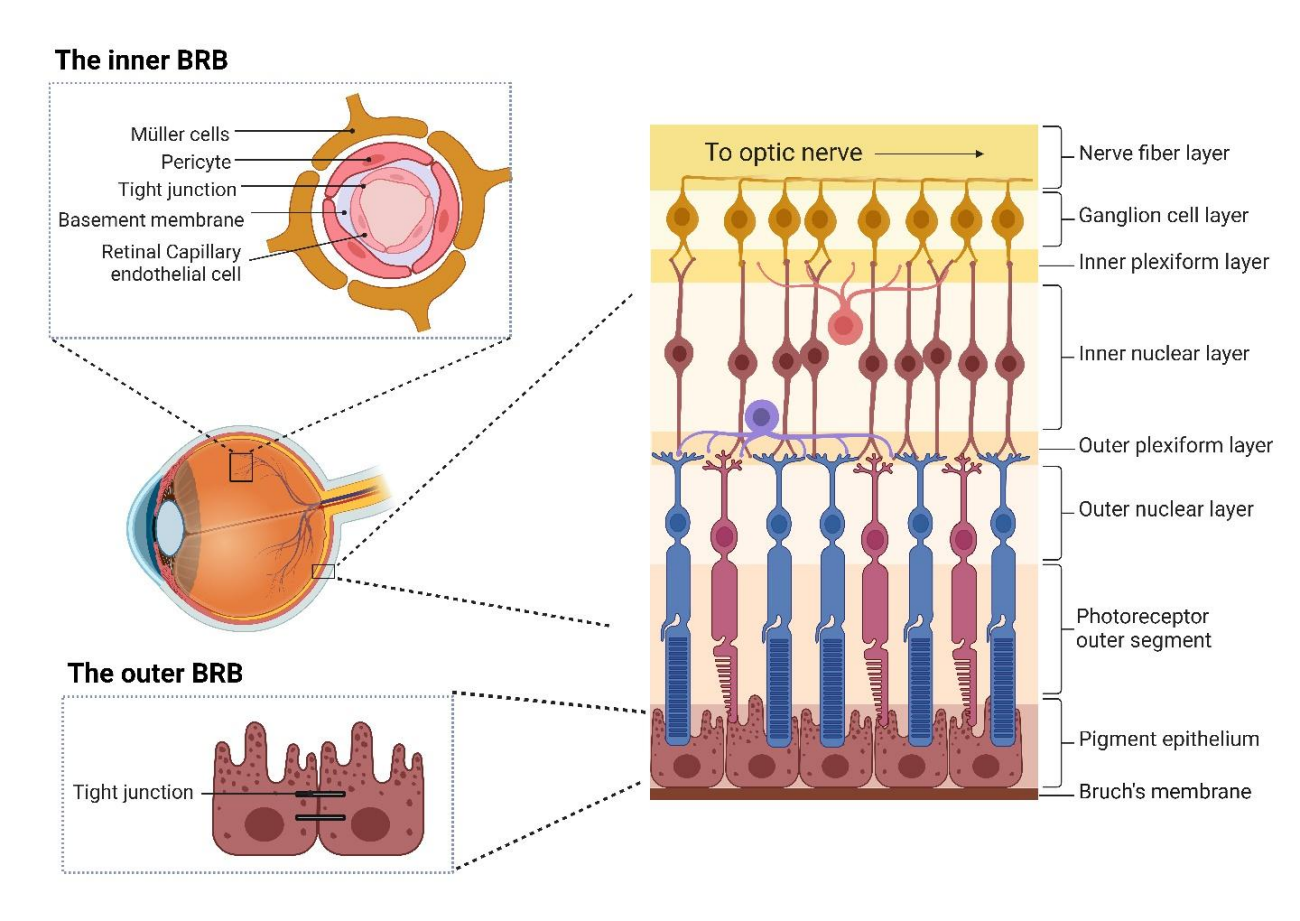
**This diagram illustrates the layered structure and the BRB of the retina**. The BRB consists of two parts: an inner barrier formed by retinal capillary endothelial cells and an outer barrier established by the RPE.

The retina, a multilayered structure at the back of the eye, is crucial for vision (Fig. 1). The outermost layer, the retinal pigment epithelium (RPE), is vital for visual cycle regulation and photoreceptor homeostasis. Adjacent to the RPE are photoreceptors (rods and cones), responsible for converting light into electrical signals. Further inward, the outer and inner nuclear layers house the cell bodies of photoreceptors and other cells like bipolar cells, while the plexiform layers contain the synapses for signal processing [15, 16]. The ganglion cell layer (GCL), located more internally within the retina, comprises retinal ganglion cells (RGCs). The axons of these RGCs converge to form the optic nerve, which carries visual information to the brain. In addition to these layers, the retina also includes Müller cells, providing structural support and regulating ion balance, and microglia, which are the retina's immune cells tasked with immune surveillance and neuroprotection [17]. A thorough understanding of this complex cellular arrangement is vital for researching retinal diseases such as RIRI.

The central nervous system (CNS), including the retina, has been traditionally regarded as an immune-privileged site due to its apparent inability to initiate an immune response. Typically, immune cells perform the function of processing and presenting antigens in the periphery [18]. To the best of our knowledge, under normal physiological conditions, there is no recorded presence of peripheral immune cells in either retinal or CNS tissues. The blood-brain barrier (BBB) serves as an anatomical and physiological shield for the CNS. Its highly selective permeability restricts the migration of immune cells and other immune mediators into the brain via tight junctions between endothelial cells, thereby preserving the unique immune privilege of the CNS [19, 20]. Consequently, the CNS appears incapable of mounting an adaptive immune response [21]. Analogously, BRB preserves retinal homeostasis, a crucial factor in maintaining the eye's immune privilege [22]. The BRB is structurally composed of two tightly connected layers: an inner barrier formed by retinal capillary endothelial cells and an outer barrier established by the RPE [22, 23]. The tight junctions serve to regulate the selective passage of ions and small solutes among adjacent cells while their receptors control the transport of smaller molecules within the CNS. This restricts the infiltration of foreign entities, such as viruses, and helps maintain homeostasis. Moreover, the RPE is highly immunosuppressive [24, 25]. Mononuclear phagocytes (MNPs), when linked to thrombospondin-1 via their CD47 receptor and reach the subretinal space, are eliminated by the RPE-expressing Fas ligand [25, 26].

However, recent studies have revealed that the CNS tissues can mount adaptive immune responses to various types of trauma, infection, or injury [27]. Despite the barriers separating the CNS and retina from immune responses, these immune components still interact with the peripheral immune system [27, 28]. Several factors influence the permeability of the BRB, allowing macromolecules and leukocytes access to the retina. These include oxidative stress, vascular endothelial growth factor, and inflammation [2, 29]. Therefore, the immune privilege of the eye, in our view, is characterized by its ability to selectively allow immune cells to infiltrate the eye for repair and healing, as needed, under various pathological conditions such as RIRI. It is not an absolute barricade preventing immune cell infiltration. Instead, it appears to be an evolutionary adaptation aimed at protecting these fragile and essential organs from irreversible damage that could be inflicted by an inflammatory attack.

**Figure 2.**
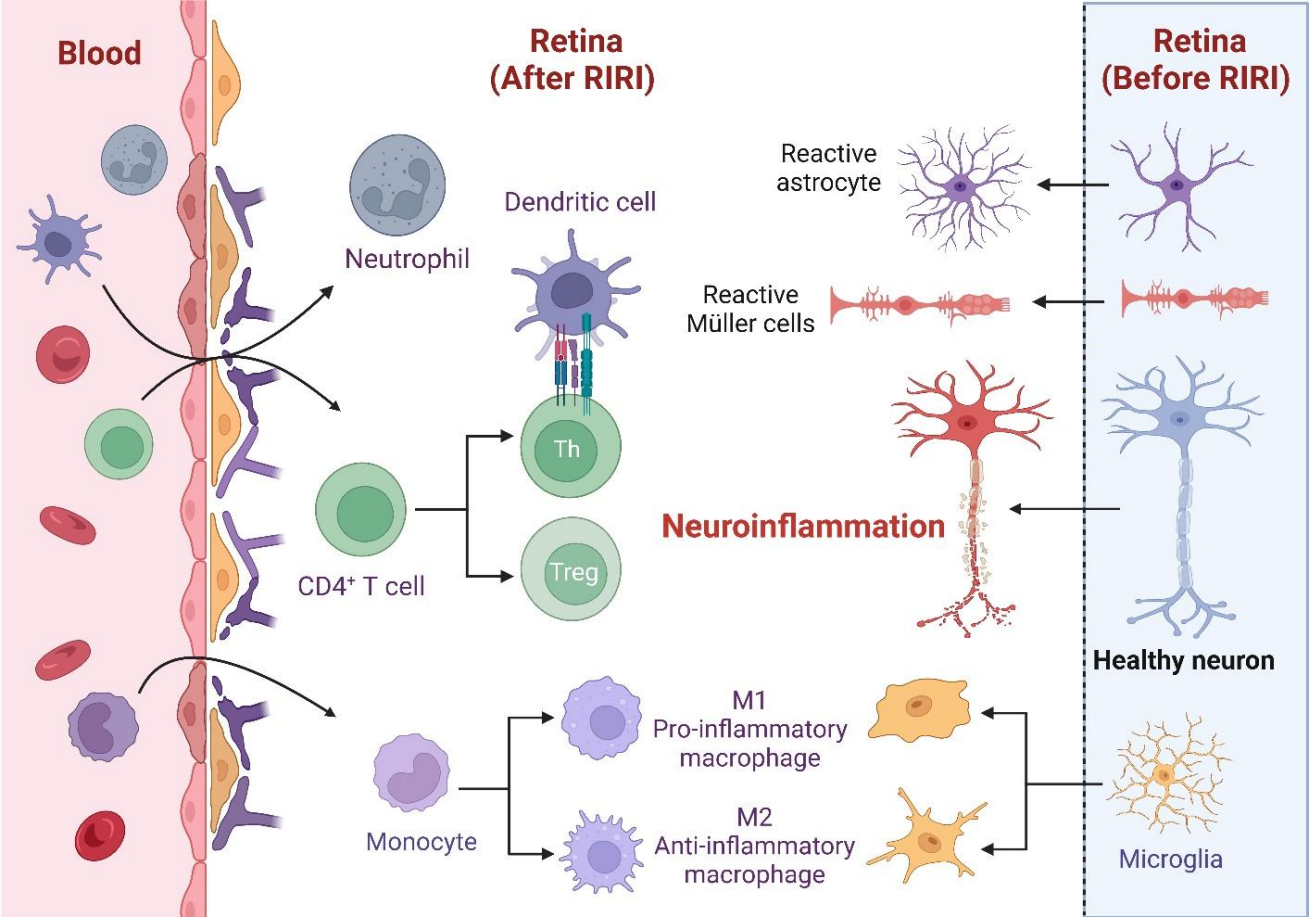
**Illustration depicting immune cell populations and their distribution within the retina**. The figure showcases immune cells, including neutrophils, CD4^+^cells, and monocytes, crossing the BRB to enter the retina. Additionally, resident immune cells within the retina, such as astrocytes, Müller cells, and microglia, are depicted. Monocytes and microglia have the potential to differentiate into M1 and M2 phenotypes.

Overall, immune responses in RIRI are regulated by both resident and peripheral immune cells. The retina is home to resident immune cells, primarily Müller cells, and also includes microglia and astrocytes-glial cells that acquire immune functions under pathological conditions (Fig. 2). Microglia, the primary immune cells in the retina, survey the retinal environment and become activated in response to injury or disease, transitioning from a ramified to an amoeboid form and releasing pro-inflammatory cytokines [30-35]. Astrocytes and Müller cells, in response to injury, undergo reactive gliosis, characterized by morphological changes, proliferation, and production of inflammatory mediators, further exacerbating the inflammatory response [36].

Peripheral immune cells, including neutrophils, monocytes, and lymphocytes, play a dynamic role in RIRI [8](Fig. 2). These cells are usually activated by ischemic injury, leading to their recruitment and infiltration into the ischemic retinal tissue, where they contribute to the inflammatory response and further tissue damage. Neutrophils, as the earliest responders, release proteolytic enzymes and reactive oxygen species (ROS), contributing to oxidative stress [8, 37]. Monocytes differentiate into macrophages upon arrival at the injury site, secreting pro-inflammatory cytokines and phagocytizing cellular debris [38]. Lymphocytes, particularly T cells, also have a significant role in modulating the immune response [39-42].

A comprehensive examination of the resident and peripheral immune responses in RIRI, detailing the complex roles and interactions of these varied cell types, is provided in the following sections. This in-depth analysis offers a deeper understanding of the immune dynamics at play in RIRI.

## 4. Resident immune cells in RIRI

Though systemic immune responses to the retina are strictly controlled because of its immune privilege, resident immune cells, including Müller cells, microglia, and astrocytes, play vital roles in maintaining immune surveillance under physiological conditions. Notably, these glial cells are seen to exhibit an early activation response in the aftermath of ischemia-reperfusion injury, underscoring their critical involvement in RIRI [43]. In the sections that follow, we will delve deeper into the roles of these resident immune cells and explore their activation patterns and responses during the course of RIRI.

### 4.1. Müller cells

Müller cells, the predominant glial cells in the retina, play a vital physiological role in maintaining retinal homeostasis and supporting the function of retinal neurons [33]. These specialized cells span the entire thickness of the retina and provide structural and metabolic support to various retinal cell types. Müller cells are involved in maintaining the integrity of BRB, regulating ion and water balance, and providing essential nutrients to neurons [44]. They also contribute to the clearance of neurotransmitters and metabolic waste products, thereby ensuring proper signaling and preventing neurotoxicity. Furthermore, Müller cells actively participate in the recycling of retinal visual pigments and help regulate the extracellular potassium concentration, which is essential for normal neuronal activity [44]. Overall, Müller cells play a critical role in maintaining retinal function and supporting the overall health of the retina.

One of the main issues in RIRI is the excessive generation of ROS during reperfusion. Retinal glia, including Müller cells and astrocytes, are responsible for maintaining redox homeostasis and have mechanisms to restore balance [45]. Müller cells are the primary glia in the vertebrate retina, spanning the entire retina and establishing connections with all types of neuronal cells in different retinal layers. As a result, they have a more critical role compared to astrocytes, which are limited to the nerve fiber layer. Following injury, Müller cells become reactive, characterized by cellular hypertrophy and hyperplasia, as well as enhanced expression of glial fibrillary acidic protein (GFAP), and contribute to the formation of glial scars. For instance, Mages et al. determined that GFAP in Müller cells in the retinas of mice is upregulated 3 days after RIRI; protein and mRNA levels also increased for 14 days [46]. Palmhof et al. also noted glial hyperplasia in Müller cells throughout the study and detected increased expression of GFAP at protein level and mRNA level at 2 h and 12 h in the retinas of rats after ischemia induction [47]. These findings suggest that Müller cells are activated in ischemic injury [48, 49]. After preconditioning, slighter GFAP immunoreactivity was observed after retinal ischemia compared with the untreated group, suggesting that the mechanism of preconditioned retinal ischemia may be related to Müller cells in the retina [50]. Interestingly, studies have shown that even under conditions of proliferative reactive gliosis induced in mice, Müller cells can still provide metabolic support to neurons [51]. Müller cells play a critical role in mitochondrial protein frataxin-mediated neuroprotection after the ischemic lesion [52]. Understanding the involvement of Müller cells in RIRI can provide insights into their contribution to the disease progression and potential therapeutic targets for mitigating retinal damage.

### 4.2. Microglia

Starting our discussion with microglia is paramount, as they constitute a crucial cell type orchestrating the immune response in RIRI. As the resident immune competent cells within the CNS, they play a crucial role in maintaining neuro-retinal homeostasis under normal conditions and responding to pathological stimuli. In the upcoming sections, we will shed light on the roles, activation patterns, and phenotypic changes of microglia in the context of RIRI. This knowledge will provide a valuable foundation for understanding the overall dynamics of the retinal immune response to ischemic insult.

### 4.2.1. Microglial role in retinal physiology

Microglia, the resident immune-competent cells within the CNS, crucially contribute to maintaining neuro-retinal homeostasis and orchestrating innate immune defense mechanisms [17, 53]. They originate from primitive macrophages of the yolk sac that populate the neuroepithelium before the formation of BBB, effectively distinguishing them from circulating macrophages [54, 55].

In the retina, microglia are primarily located in three layers: the retinal nerve fiber layer (RNFL), the inner plexiform layer (IPL), and the outer plexiform layer (OPL). Microglia in the GCL, the innermost part of the retina, reside around the cell bodies of RGCs or their axon projections. It is important to note that most microglia are positioned in the IPL and OPL. Although microglia in the IPL and OPL share specific attributes, like the presence of the colony-stimulating factor-1 receptor (CSF1R) crucial for their survival, key distinctions in homeostasis between these two populations have been unveiled in recent studies. Microglia in the IPL primarily rely on IL-34, an alternative ligand of CSF1R produced by RGCs, for their maintenance [56-58], whereas those in the OPL do not. Further, these two microglial pools contribute differently to visual processing: IL-34-dependent microglia in the IPL have a significant role in cone bipolar cell responses, a function not seen in the OPL [59].

Microglia continually monitor their surroundings in the retinal microenvironment, maintaining functional equilibrium by interacting with other retinal cells [59]. When disrupted, activated microglia can destroy degenerated neurons and photoreceptors via phagocytosis, potentially exacerbating retinal damage by releasing numerous proinflammatory mediators. As the immune gatekeepers of the retina, microglia defend against harmful stimuli, facilitate tissue repair, and regulate immune responses through a repertoire of mechanisms, including phagocytosis, complement activation, antigen presentation, and inflammation involvement. Notably, most microglia in a healthy retina do not express MHCII classes or the costimulatory factors necessary for antigen presentation, with the absence of MHCII expression in naive retinal microglia also observed by O'Koren et al. and Zhang et al. [33, 60]. Research into the antigen-presenting capabilities of mouse retinal microglia has likewise demonstrated that these cells have a limited capacity to serve as antigen-presenting cells (APCs) [61]. Phagocytosis is a key function of retinal microglia, as they partake in synaptic pruning, elimination of invading microbes, clearance of cell debris, and removal of apoptotic cells, all of which can influence the progression of retinal diseases. Moreover, microglia are known to express a variety of receptors, such as TLRs, Fc receptors, complement receptors, αvβ3 and αvβ5, transmembrane protein 119 (TMEM119), triggering receptor expressed on myeloid cells 2 (TREM2), chemokine receptor, Mer tyrosine kinase(MerTK), and scavenger receptor [62].

### 4.2.2. Activation and proliferation of microglia

Resting microglia, typically exhibiting a highly ramified morphology, contribute to maintaining retinal homeostasis through immune surveillance [63-65]. In response to injuries or disease, these microglia become activated, undergoing phenotypic and functional changes as part of an innate immune mechanism to shield the retina from infection or damage. However, overactivation of microglia can lead to the release of inflammatory cytokines, the recruitment of inflammatory effector cells, and the exacerbation of inflammation [66, 67]. This process results in a pro-inflammatory retinal microenvironment and a compromised BRB, allowing lymphocyte infiltration [68-70]. These events are thought to precede neuron death [71, 72].

While invading monocytes may share phagocytic capabilities, microglia are considered more efficient phagocytes and are responsible for a substantial portion of debris removal, as observed in brain ischemia-reperfusion (IR) injury and spinal cord injury models [73, 74]. However, the commonly used microglial marker Iba-1 does not distinguish between microglia and invading monocytes. Upon activation, microglia/macrophages undergo characteristic morphological changes—from a ramified state in rest to an amoeboid-like state—and upregulate specific activation markers. Notably, levels of markers such as CD16, CD86, and CD206 peaked on day 7 post-RIRI. Additionally, these cells release pro-inflammatory cytokines (including TNF-a, IL-1β, IL-6, and IFN-g) and downregulate anti-inflammatory factors, such as transforming growth factor-β (TGF-β) [1].

Post-injury, microglia likely phagocytose dying RGCs and displaced amacrine cells (dACs) in the GCL, as well as synapses in the inner plexiform layer IPL, thereby removing dead neurons and debris [8]. Abcouwer et al. suggested that microglia proliferated and migrated to the GCL and IPL at day 4, slowly returning to near-normal population sizes within four weeks [75]. Approximately half of the Tunel-positive cells in the GCL expressed the RGCs marker RBPMS. This is consistent with the fact that about half of the cells in the GCL are RGCS, and the remainder are dACs [76].

Laquinimod [77] and Baicalein [78] have been shown to protect RGCs by inhibiting the activation of microglia. Inhibition of microglial activation and subsequent T cell recruitment via intravitreal injection of a CSF-1R neutralizing antibody (CSF-1RAb) post-RIRI preserves retinal immune homeostasis without depleting the microglia. This supports the concept that early activation of microglia and macrophages is crucial to the initiation of CD4^+^T cell responses post-RIRI. However, further research is needed to understand the interactions between microglia and other immune cells after RIRI.

Interestingly, microglia/macrophages can shift functionally between pro-inflammatory M1 and anti-inflammatory M2 phenotypes, depending on the local environment—a process referred to as polarization [79]. M1 phagocytes contribute to neuronal degeneration and neural network dysfunction by producing various pro-inflammatory cytokines and mediators [79]. In contrast, M2 phagocytes inhibit inflammation and promote tissue remodeling by altering gene expression and producing neuroprotective factors [79, 80]. An imbalance in M1 and M2 phenotype distribution, particularly a deficiency in the M2 phenotype, is associated with neurodegenerative conditions such as ischemic stroke, traumatic brain injury, and spinal cord injury [81, 82], and similar observations have been made in RIRI [8, 75]. Therefore, therapeutic strategies aiming to promote M2 phenotype over M1 may be effective in mitigating ischemia damage. For example, intravitreal injection of minocycline treatment has been shown to promote M2 polarization of microglia/macrophages, alleviating neuron loss induced by RIRI [75].

In summary, microglia are vital in maintaining retinal homeostasis responding to injury by activation and functional transformation. Overactivation, however, can lead to damage and a pro-inflammatory environment. Post-injury, these cells remove debris and adjust their number and location, showing great adaptability. Nonetheless, an imbalance in their pro-inflammatory and anti-inflammatory states can lead to neurodegeneration, emphasizing the importance of their proper regulation for retinal health.

## 4.3 Astrocytes

### 4.3.1 Physiological role of astrocytes

Astrocytes, being the most prevalent cell type in the CNS, fulfill numerous vital functions within the retina [83]. They primarily offer metabolic assistance to neurons. In doing so, they bolster the health and longevity of RGCs, the axons of which constitute the optic nerve. Astrocytes provide essential nutrients to RGCs and assist in preserving the equilibrium of ions and neurotransmitters in the retinal milieu [84]. Additionally, astrocytes play a part in establishing and preserving BRB, a discerning partition akin to BBB, overseeing the transfer of elements between the retina and bloodstream [85]. This role is paramount for maintaining the strictly regulated surroundings vital for the translation of visual signals.

Furthermore, astrocytes have a role in synaptic operations and adaptability [86]. They can adjust neurotransmitter absorption and discharge, consequently influencing synaptic connectivity and the formation of neural circuits. They also liaise with other glial cells, such as microglia, playing a part in the immune reaction and overall stability of the retina. Astrocytes also have an antioxidant defense function, producing a variety of antioxidant enzymes to counter oxidative stress, which could harm retinal neurons [82].

In adverse conditions, like ischemic injury or glaucoma, astrocytes undergo what is termed reactive gliosis [82]. This is a protective mechanism intended to shield the retina from further damage. However, excessive or sustained gliosis can, in certain scenarios, intensify the harm, leading to retinal deterioration [87]. The multifaceted functions of astrocytes emphasize their significance in upholding retinal well-being and operations.

### 4.3.2 Astrocytes in RIRI

Astrocytes play a significant role in RIRI, as their dysfunction and damage can lead to subsequent neuronal death. Conversely, preserving the activity and function of astrocytes has a protective effect on neurons in the GCL [88, 89]. Activation of microglia has been shown to trigger astrocyte activation and reactive gliosis, contributing to neuron loss [90]. Following ischemia-reperfusion injury, the number of astrocytes surrounding the damaged area increases and undergoes significant activation, a phenomenon known as reactive gliosis characterized by hypertrophy, hyperplasia, and enhanced expression of GFAP [91-93]. However, prolonged and severe reactive gliosis may exacerbate neuronal degeneration and apoptosis.

Inflammatory processes involving astrocytes contribute to the pathogenesis of RIRI. Pro-inflammatory cytokines, such as TNF-α and IL-1β, are expressed in astrocytes following ischemia-reperfusion injury, promoting cell death [94-96]. TNF-α signaling through TNF receptor 1 triggers programmed cell death signaling while also inducing pro-survival effects through TNF receptor 2, both of which are expressed by RGCs post-injury and differentially regulate pro-survival and pro-apoptotic pathways [97]. IL-1β signaling through the IL-1 receptor (IL-1R) facilitates the long-lasting activity of NF-κB and upregulates various inflammatory molecules, including cytokines, chemokines, and CAMs [96, 98, 99]. Chemokines, such as CC and CXC members, attract peripheral immune cells following RIRI, with CXCR8 primarily attracting neutrophils and CCR2 acting as a monocyte attractant [88]. Astrocytes and endothelial cells express increased levels of intercellular CAM-1, p-selectin, and vascular CAM after RIRI, which is promoted by NF-κB activation of astrocytes [88]. Blocking these molecules has been shown to relieve inflammation, reduce cell death, and inhibit the invasion of peripheral immune cells. Additionally, the involvement of Cyclooxygenase 2 (COX-2), an enzyme responsible for prostaglandin synthesis, and its downstream product, Prostaglandin E2 (PGE2), in RIRI has been observed [100-105]. The COX-2/PGE2/EPs pathway has been implicated in the neurodegenerative reaction and secondary injury of RGCs, highlighting the role of astrocytes in regulating the inflammatory response in RIRI [106]. Furthermore, astrocytes contribute to oxidative stress in RIRI through the transcriptional regulation of phagocyte NADPH oxidase (PHOX) by NF-κB [88, 107]. PHOX, highly expressed in the GCL and inner nuclear layer (INL) of the retina, plays a significant role in RIRI [108, 109]. Animals with genetically blocked gp91PHOX subunit, a component of PHOX, exhibited reduced oxidative stress, reduced ROS levels, and a higher number of neurons in the GCL [107]. Activation of NF-κB by TLR3 signaling was also found to promote significant cell death in RIRI [36].

In conclusion, astrocytes in RIRI are involved in both inflammatory responses and oxidative stress, contributing to neuronal damage and loss. Understanding the mechanisms underlying astrocyte-mediated inflammation and oxidative stress is essential for developing therapeutic strategies aimed at protecting retinal neurons and mitigating the effects of RIRI.

### 4.4. Role of pattern recognition receptors in inflammatory pathways in RIRI

Inflammation, a key defense mechanism of our body, involves immune cells releasing various substances like cytokines and chemokines. These substances act like messengers that alert the body to respond to injuries or infections. RIRI can induce early immune responses, including innate immunity, adaptive immunity, and inflammation.

The innate immunity system, through pattern recognition receptors (PRRs), recognizes biomacromolecules of pathogen-associated molecular patterns (PAMPs) or damage-associated molecular patterns (DAMPs) [110]. Think of PRRs as the body's alarm system that detects danger and triggers a series of signals to defend itself. PAMPs, like lipopolysaccharide and dsDNA, originate from pathogens, while DAMPs emerge from necrotic or apoptotic cells, including proteins such as HMGB1 and mitochondrial components [111, 112]. Inflammasomes, intricate intracellular protein complexes, assemble upon detecting PAMPs or DAMPs via specific PRRs. Toll-like receptors (TLRs) and NOD-like receptors (NLRs) are representative PRRs, expressed by various immune and non-immune cells, detecting external and internal damage signals, sparking the innate immune response [113, 114]. The activation of PRRs leads to the formation of multiprotein inflammasome complexes that serve as platforms for the activation of caspases. These caspases can activate the nuclear factor kappa-B (NF-κB) signaling pathway, promoting the maturation and secretion of inflammatory cytokines and amplifying the immune response [115].

When microglia suffer from damage, such as oxidative injury, activated microglia upregulate TLRs in the CNS [116]. In previous studies, it was suggested that TLR2 is involved in retinal function in DR and uveitis [117, 118]. In the early stages of RIRI, activation of the TLR2/MyD88/NF-κB pathway leads to the release of pro-inflammatory cytokines such as IL-1β, IL-16, TNF-α, TGF-β. The TLR3 medicated Myd88-independent pathway was also activated. However, retinal ischemia did not induce TLR2 expression at the mRNA and protein levels. The inconsistent results may be related to the different animal models used [35]. TLR4 is closely related to the activation of inflammasomes and cell death, which seems to be more important than other TLRs in RIRI. As a specific accessory protein, Myeloid differentiation protein 2 induced RIRI through the TLR4 pathway, contributing to the accumulation of ROS in vivo and in vitro [119]. Various agents, including Pioglitazone, Puerarin, N-acetylserotonin, and Dexmedetomidine, can promote retinal ganglion cell survival by suppressing the TLR4/NF-κB pathway in RIRI mice [120-123].

### 4.5. The complement system takes part in RIRI

The complement system, a part of the innate immune system, is activated in RIRI and plays a dual role in immune responses [124]. Complement activation can occur through the classical, lectin, and alternative pathways, converging at the central component C3 [125, 126]. Activation of the complement system leads to immune defense and inflammation, including the recruitment and activation of immune cells and opsonization of pathogens [127]. In RIRI, complement components such as C1q and C3 are upregulated, coinciding with microglial activation and increased glial cell density in the retina [32, 128]. Understanding the mechanisms of complement system involvement in RIRI can provide insights for developing therapeutic strategies to mitigate its detrimental effects.

Complement activation in response to CNS injury has both beneficial and harmful consequences, as it facilitates the rapid removal of dying cells and cellular debris, limiting the extent of local inflammatory responses [129]. Inhibition of the complement cascade by removing specific complement components has been shown to reduce neuronal damage in hypoxic-ischemic brain injury [130-132]. Studies have indicated a correlation between RIRI and the expression of complement components C1q and C3 in the retina of humans with IOP and RIRI rats [128]. In RIRI, there is a significant upregulation of C1q expression in the retina as early as 72 hours after the injury [32]. This upregulation of C1q coincides with the activation of microglia and astrocytes, as well as a notable increase in the density of glial cells in the retina [32]. C1q serves as a primary mediator of microglial activation, as demonstrated by studies showing that C1qa-knockout completely prevents microglial activation and density changes in vivo, although it does not affect astrocytes [32].

The involvement of the complement system in RIRI highlights its dual role in immune responses, including the activation of glial cells. Further research is needed to fully understand the precise mechanisms by which the complement system contributes to retinal ischemic injury and to explore its potential as a therapeutic target for mitigating the damage caused by RIRI.

### 4.6. Inflammasomes in RIRI

**Figure 3.**
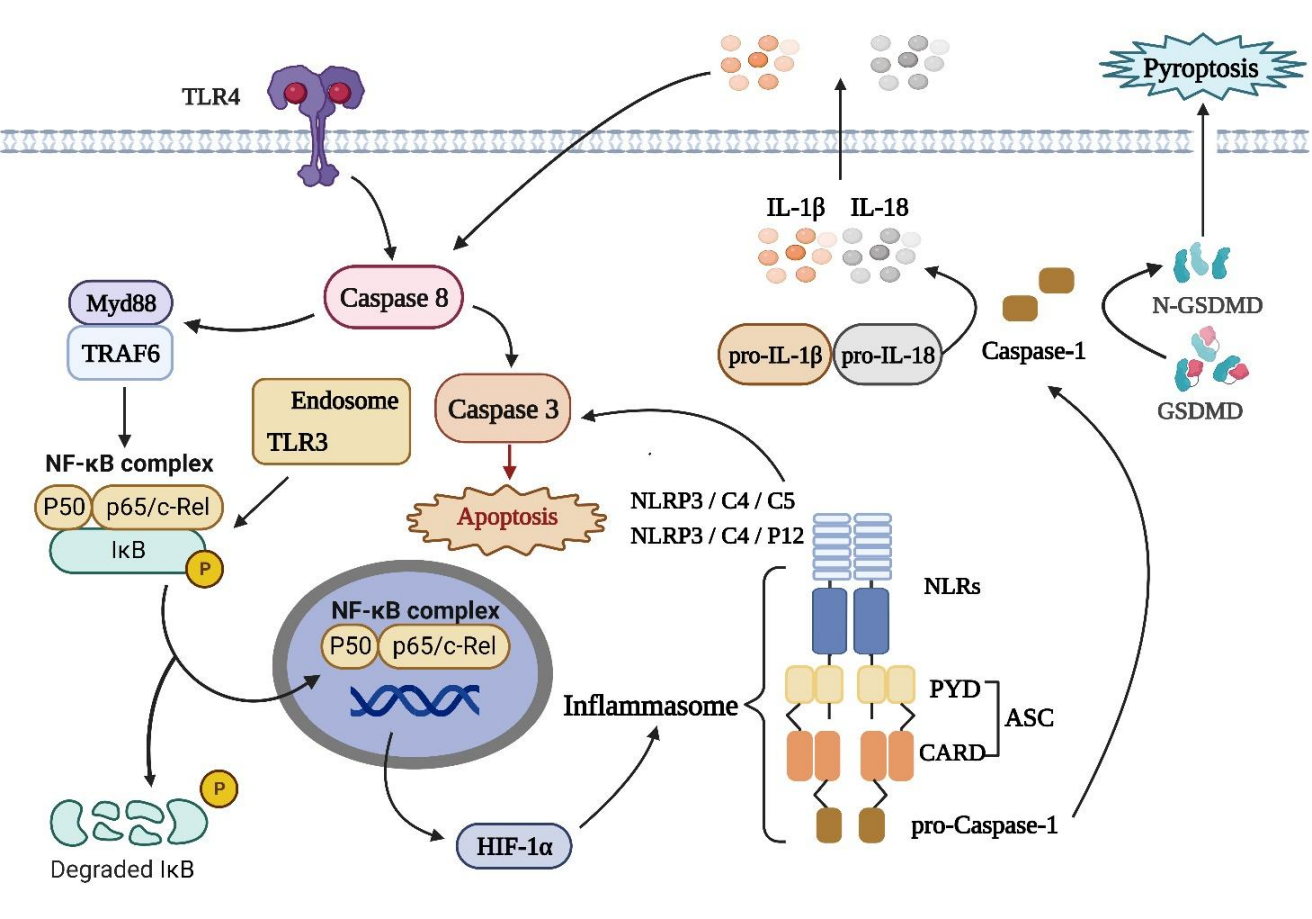
**TLR4 signaling and cellular outcomes**. This figure depicts how TLR4 triggers the activation of inflammasomes through distinct signaling pathways, ultimately leading to cell death. TLR4 activates different inflammasomes through the NF-κB pathway, which in turn activate caspase-1 and release pro-inflammatory factors IL-1β and IL-18 secretion, leading to pyroptosis. TLR4 can also induce apoptosis by activating caspase-8 and downstream effector caspase-3.

Recent research highlights the critical role of inflammasomes, which are like security alarm systems in cells, in starting and driving the progression of RIRI. (Fig. 3). Inflammasomes are multimeric protein complexes that assemble in response to danger cues, including PAMPs and DAMPs. These conglomerates, consisting of NLR family members such as pyrin domain-containing 1 (NLRP1), pyrin domain-containing 3 (NLRP3), CARD domain-containing protein 4 (NLRC4), CARD domain-containing protein 5 (NLRC5), and pyrin domain-containing 12 (NLRP12), have been implicated in various inflammatory mechanisms associated with RIRI [14, 133, 134].

During inflammasome activation, TLR4 is a critical component of the innate immune system that plays a crucial role in recognizing microbial components. When TLR4 binds to its ligands, such as PAMPs or DAMPs, it initiates a series of signaling events that lead to various cellular responses [123]. Caspase-8 plays a dual role in TLR4 signaling by regulating both MyD88-dependent and TRIF-dependent pathways (Fig. 3). It achieves this by cleaving MyD88 and TRIF, respectively, leading to the activation of downstream signaling cascades that are essential for the innate immune response against pathogens and the induction of pro-inflammatory and antiviral genes [110]. In addition, once activated, caspase-8 initiates apoptotic pathways for eliminating infected or damaged cells and maintaining tissue homeostasis [133, 134]. Concurrently, TLR4 signaling, primarily via the MyD88-dependent pathway, activates the transcription factor NF-κB in RIRI rats [35]. This event results in the translocation of NF-κB from the cytoplasm to the nucleus, where it initiates the transcription of pro-inflammatory genes. The activation of NF-κB can also induce the expression of hypoxia-inducible factor 1-alpha (HIF-1α). HIF-1α is a central regulator of cellular responses to hypoxia and plays a crucial role in orchestrating the assembly of an intracellular multi-protein complex known as the inflammasome. The inflammasome typically comprises proteins like ASC, NLR, and procaspase-1 [134]. Activation within the inflammasome complex leads to the activation of caspases, which contribute to the processing of pro-inflammatory cytokines, including IL-1β and IL-18. Secretion of IL-1β and IL-18 following inflammasome activation triggers an inflammatory response by recruiting immune cells and amplifying the immune reaction (Fig. 3). In some circumstances, inflammasome activation can also culminate in pyroptosis, a specialized form of programmed cell death characterized by cell swelling and membrane rupture, primarily mediated by gasdermin D (GSDMD) [135-137].

As early as 2011, studies found that HMGB1 can be released from necrotic cells and induce inflammatory responses and RGC loss after RIRI [138, 139], and the TLR4/NF-κB/NLRP3 pathway is the crucial mechanism [140-142]. HMGB1 significantly activates TLR4 in RGCs and glial cells after RIRI, leading to the upregulation of caspase-8 and activation of NLRP3, which induces the mature of IL-1β through a caspase-1- and caspase-8-dependent pathway and the death of RGCs [133, 134]. The intravitreal injection of an anti-HMGB1 monoclonal antibody can reduce corresponding inflammatory signaling and provide RGC neuroprotection [143]. Additionally, NLRP12 collaborates with NLRP3 and NLRC4 to induce IL-1β processing and pyroptosis, mediated by caspase-1-dependent GSDMD cleavage [14]. Caspase-8 promoted NF-kB translocation to drive HIF-1α signaling to initiate the pyroptosis mentioned above [14]. The mature form of IL-1β, a critical mediator of neuroinflammatory cascades in RIRI, initiates various NLR-dependent pathways, amplifying the inflammatory response [14]. Furthermore, NLRC5 binds directly to NLRP3 and NLRC4 in inflammasomes, contributing to microglial pyroptosis and apoptosis, which mediate retinal ischemic damage [144]. The dysregulation of NLRP3/6 inflammasomes, as well as the involvement of long non-coding RNA H19, has also been implicated in RIRI-induced microglial pyroptosis and neuronal death [34]. However, further investigations are needed to elucidate the precise molecular mechanisms underlying the interplay between pyroptosis and apoptosis pathways and their collective contribution to RGC death in RIRI. Understanding the role of inflammasomes in RIRI pathogenesis holds promise for the development of targeted therapeutic strategies aimed at mitigating retinal damage and preserving visual function.

Taken together, inflammasomes have emerged as pivotal players in RIRI. TLR4 serves as a key initiator, detecting danger signals. Caspase-8 has a dual role, cleaving MyD88 and TRIF to trigger innate immune responses, while TLR4 activates NF-κB, promoting pro-inflammatory gene expression and HIF-1α. This orchestrates inflammasome formation, resulting in caspase activation, pro-inflammatory cytokine production, and, in some cases, pyroptosis. Understanding these processes offers the potential for targeted therapies to protect the retina and preserve vision in RIRI.

## 5. Peripheral immune cells in RIRI

Peripheral immune cells play a crucial role in the pathophysiology of RIRI [8]. Following reperfusion, a cascade of events is triggered in the retina, leading to the activation and recruitment of immune cells from the bloodstream to the damaged site. Neutrophils, monocyte-derived macrophages (MDMs), and T cells are among the key peripheral immune cells involved in the immune response after RIRI [8]. Neutrophils are the first responders, migrating to the ischemic retina and potentially contributing to tissue damage through the release of matrix metalloprotease-9 (MMP-9) [37]. MDMs participate in the clearance of dead cells, tissue repair, and inflammatory processes [78, 145]. T cells, such as CD4^+^ T cells, exhibit both pro-inflammatory and regulatory functions, influencing the immune response and neuronal damage in RIRI [39, 40]. Understanding the roles of these peripheral immune cells and their interactions with retinal cells is crucial for elucidating the mechanisms underlying RIRI and developing effective therapeutic strategies [146].

### 5.1 Neutrophils

Neutrophils, as one of the key immune cells involved in the innate immune response after RIRI, rapidly infiltrate the retina as part of the initial immune response to neuroinflammation. However, the exact role of neutrophils in RIRI remains poorly understood. Studies have shown that ischemic infiltration of neutrophils occurs in animal models of stroke, and the number of circulating neutrophils increases in patients with stroke, correlating with stroke severity, infarct size, and functional prognosis [147]. High neutrophil-lymphocyte ratios have been associated with poor neurological recovery after stroke, indicating a potentially detrimental role of neutrophils in this condition. Glaucoma patients present a high blood neutrophil-lymphocyte ratios positively correlating with the degree of damage [148]. Under certain stimuli, neutrophils extrude a DNA meshwork and cytosolic granule proteins, known as a neutrophil extracellular trap (NET) [149]. The NET formation is accompanied by the cell death called NETosis [150]. Glaucoma patients also have neutrophil infiltration and cytokine overexpression that promote the process of trabecular meshwork NETosis [151, 152]. High levels of lipocalin 2 (LCN2), a neutrophil protein, presents in the aqueous humor of glaucoma patients and RGCs of glaucoma animal models [153, 154]. Furthermore, Feng and Xu suggested LCN2 as a biomarker for glaucoma [155]. Neutrophils release MMP-9, which can lead to BBB disruption, extracellular matrix degeneration, and increased neuroinflammation [156, 157]. These findings suggest that the infiltration and accumulation of neutrophils may contribute to ischemic brain injury. Similarly, neutrophils are also implicated in RIRI. MMPs play a critical role in maintaining the integrity of BRB, and blocking or inhibiting MMPs may represent a novel therapeutic strategy to improve the prognosis of RIRI [158]. Recent studies have revealed that neutrophils exhibit functional plasticity similar to other immune cells, such as macrophages. Neutrophils can exhibit two functional phenotypes: pro-inflammatory N1 neutrophils and alternative anti-inflammatory N2 neutrophils [159-161]. The ratio of N1 to N2 neutrophils in RIRI may be linked to the severity of retinal damage, although further research is required to validate this relationship. Understanding the precise role and functional heterogeneity of neutrophils in RIRI is essential for elucidating their contribution to retinal injury and for developing targeted therapeutic interventions [159-161].

### 5.2 Monocyte-derived macrophages

MDMs play a significant role in RIRI. The retina contains a diverse population of MNPs that continuously surveil the neuronal parenchyma and border tissues. This includes resident microglia within the parenchyma and border-associated macrophages, such as long-lived retinal perivascular macrophages and short-lived choroidal macrophages adjacent to the retina [59, 78, 162-164]. Recent studies have provided more definitive evidence that microglia and monocytes represent distinct lineages of MNPs with distinct functions and responses to injury [78, 145, 165]. Similar to the brain and spinal cord, retinal ischemia leads to large-scale recruitment of monocytes and subsequent differentiation into macrophages, contributing to the MDMs response [163, 166, 167].

The involvement of macrophages in the retina has been highlighted in studies investigating optic nerve injury. For instance, Zeng et al. utilized optical coherence tomography (OCT) to visualize and analyze macrophage-like cells (MLCs) in patients with acute nonarteritic central retinal artery occlusion (CRAO). MLCs consist of microglia, perivascular macrophages, MDMs, and hyalocytes from the vitreous. Increased density and morphological changes of MLCs were observed after acute retinal ischemia-reperfusion [168, 169]. After acute RIRI in CRVO, The increased density and morphological changes of MLCs may suggest the aggregation and activation of MLCs, which are correlated with the disease course and the ischemic severity [169]. Moreover, macrophage activation following acute RIRI was found to have detrimental effects on the health of endothelial cells in the retina [170]. Additionally, the responses of macrophages can differ depending on the immune background, leading to varying effects on the loss of RGCs [170].

Specific surface markers influence the differentiation and functional phenotypes of monocytes. Monocytes are bone marrow-derived myeloid cells in the blood that can be classified into three main subsets: classical, intermediate, and non-classical, based on the expression of markers such as CD14, CD16, Ly6C, CCR2, and CX3CR1 [171-173]. Classical monocytes (CD14^+^CD16^-^ in humans, Ly6C^+^CCR2^high^CX3CR1^low^ in mice) are recruited to inflamed tissues and differentiate into pro-inflammatory macrophages that release ROS and pro-inflammatory cytokines, including TNF-α, IL-1β, and IL-6, which can contribute to retinal cell damage [174-179]. In contrast, non-classical monocytes (CD14^-^CD16^+^ in humans, Ly6C^-^CCR2^low^CX3CR1^high^ in mice) are recruited to non-inflamed tissues and differentiate into anti-inflammatory macrophages that release cytokines such as IL-4, IL-10, and IL-13, playing a crucial role in reparative processes [180, 181].

The role of MDMs in RIRI is complex and context-dependent. While macrophages can be beneficial in the early stages of injury by facilitating the clearance of dead cells and promoting tissue repair, excessive macrophage activation can lead to chronic inflammation and tissue damage. Further research is needed to fully understand the underlying mechanisms and functions of MDMs in RIRI, which could potentially lead to the development of new treatments for retinal diseases.

### 5.3 T cells

In recent years, the role of adaptive immunity, particularly T lymphocytes, has been recognized as necessary in immune responses following RIRI. CD4^+^ T cells can be classified into two subgroups based on their functions: T helper cells (Th) and T regulatory cells (Tregs). Th cells recognize exogenous antigenic peptides presented by major histocompatibility complex class II (MHCII) molecules on APCs. Depending on the cytokine environment, Th cells can differentiate into either Th1 or Th2 cells, which enhance cellular immunity and humoral immunity, respectively [182].

Studies have shown that the absence of T and B lymphocytes in severe combined immunodeficient (SCID) mice led to a higher survival rate of RGCs in a model of high IOP [39]. Replenishing CD4^+^ T cells alone in SCID mice reversed the preservation of RGCs. Further investigations have demonstrated that elevated IOP can trigger the infiltration of CD4^+^ T cells in the retina. Chen et al. identified heat shock proteins (HSPs) as pathogenic antigens for these T cells, contributing to the development of RGCs and axon loss over an extended period in glaucomatous mice and human glaucoma patients [40].

The balance between Th1 and Th2 cytokine production influences various pathological processes and can play both causative and protective roles in neuronal damage [183, 184]. Mice with defective Th2 helper T cells, due to dysfunctions of the signal transducer and activator of transcription 6 (STAT6), exhibited significant resistance of RGCs to cell death [39]. These findings support the critical role of adaptive immunity, particularly Th2 cells, and their microenvironment in neuronal damage following RIRI.

T regulatory cells (Tregs), characterized by the expression of the fork-head box P3 (Foxp3) transcription factor, are capable of regulating the inflammatory environment and immune homeostasis [185]. Tregs can migrate to the site of injury and mitigate inflammation by increasing the levels of anti-inflammatory factors and activating macrophages to clear debris [127, 186, 187]. While Tregs are primarily derived from the thymus (thymus-derived Tregs or tTregs), CD4^+^ cells at the site of injury can be reprogrammed and acquire Treg phenotypes, referred to as peripheral Tregs (pTregs), which contribute to immune tolerance during tissue injury [188]. Agrawal et al. demonstrated the reprogramming of CD4^+^ T cells into Tregs in vitro through intravitreal injection of mesenchymal stem/stromal cells in RIRI mice, resulting in reduced retinal neuroinflammation and improved visual function [41]. These findings highlight the importance of T cell-mediated adaptive immune responses in ischemia-induced nerve damage, and the establishment of an immunosuppressive environment may represent a potential therapeutic target for retinal ischemic diseases.

## 6. Interplay of resident immune cells and peripheral immune cells and their potential relationship with cytokines and signaling pathways

As we have reviewed, the immune system plays a pivotal role in RIRI, with both peripheral and resident retinal immune cells contributing significantly to the disease pathology. These cells are fundamental to inflammatory responses, with resident immune cells maintaining immune surveillance under normal conditions and responding to pathological changes and peripheral immune cells migrating to the site of injury upon activation.

There are cross-talks between resident immune cells in the retina. The activation of retinal microglia and Müller cells leads to the continuous secretion of inflammatory mediators such as TNF-α and IL-1β, which aggravates excitatory neurotoxicity [84, 189, 190]. Schultz et al. showed that frataxin overexpression in Müller cells improves RGC survival with a decreased microglia response after RIRI [52]. Using a coculture system, Tezel and Wax found that glial cells secrete TNF-α under simulated ischemic conditions, promoting the direct death of RGCs [191]. Transwell assay showed that the supernatants of primary RGCs that experienced more death attracted a larger number of microglia and Müller cells, suggesting that cytokines released by damaged RGCs promote the aggregation of microglia and Müller cells, which in turn aggravates inflammation and RGC death [192]. Qin et al. used multiple cell death inhibitors(inhibitors of apoptosis, necroptosis, and ferroptosis: z-VAD-FMK, Necrostatin-1, and Ferrostatin-1, respectively) in RIRI to inhibit the activation of phagocytes and reduce the elevation of inflammatory mediators, while weakening the chemotaxis of phagocytes to RGCs in vivo [192]. Therefore, the interaction between Glial Cells and RGCs after RIRI aggravates retinal damage in a vicious circle.

However, the upregulation of PEDF, VEGF, and IL-6 in RGCs can foster the neuroprotective status of Müller cells and prevent neuronal damage caused by ischemic injury [193, 194]. RGCs can enhance their own protection by influencing the release of neuroprotective factors from Müller cells through co-cultures [193]. PEDF, VEGF, and IL-6 are essential for protecting RGCs from apoptosis and stimulating their release through bidirectional communication between RGCs and Müller cells may enhance neuronal survival [193]. Thus, because of intimate glial-neuronal contacts, the effect between retinal glial cells and RGCs may be bidirectional based on released diffusible mediators. Nevertheless, whether this interaction is beneficial or harmful to retinal damage is still uncertain, and further research is needed.

**Figure 4.**
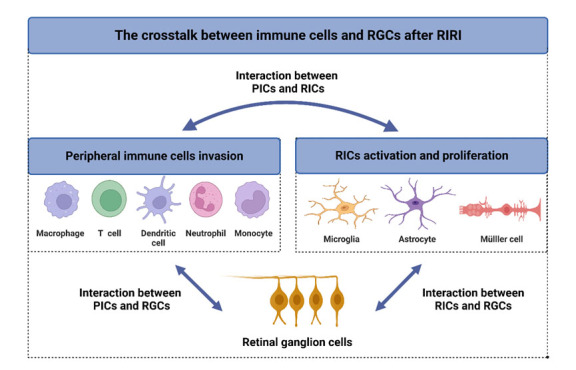
**Schematic representation of the crosstalk between immune cells and RGCs following RIRI**. The figure illustrates the complex interactions between Peripheral immune cells (PICs) and resident immune cells (RICs) within the retina and their connection with RGCs, demonstrating the intricate relationship between immune cells and neurons in response to ischemia.

In addition, there is a complex interplay between peripheral immune cells and resident immune cells during RIRI (Fig. 4). Activated microglia and reactive glial cells release chemokines that attract peripheral immune cells to the injury site, amplifying the inflammatory response. Conversely, infiltrating peripheral immune cells can further activate resident immune cells, creating a feedback loop that exacerbates inflammation and tissue damage. Understanding the precise roles and interactions of these immune cell populations during RIRI is crucial for developing targeted therapies that could modulate these cells' responses to mitigate inflammation and protect the retina from ischemia-reperfusion injury.

The process of RIRI ignites an intricate cascade of events involving various immune cells. Immediately following the injury, an acute inflammatory phase is initiated, marked by the disruption of BRB and the activation of vascular endothelial cells. These endothelial cells, in turn, increase the expression of selectins and cell adhesion molecules (CAMs) as well as enhance their permeability [8, 195]. During this phase, there is a marked reduction in retinal cells, accompanied by the activation of microglia and the significant infiltration of blood-derived immune cells such as monocytes, macrophages, neutrophils, T cells, and DCs [196]. This leukocyte extravasation is integral to the inflammatory response, involving multiple steps from chemoattraction and rolling adhesion to tight adhesion and transmigration [197-199]. By the first day post-injury, a surge of CD45^+^ leukocytes can be detected within the vessel lumen, suggesting the initiation of leukocyte extravasation [8]. In response to RIRI, chemotactic agents are produced within the retinal vascular system, subsequently spreading to the inner layer of the retina. These chemokines potentially orchestrate the homing and directed migration of leukocytes into the inner retina, leading to localized retinal damage, primarily in RGCs and other inner neuronal structures [200].

The reparative process post-RIRI is marked by an intense initial inflammatory phase, with granulocyte (neutrophil) and pro-inflammatory monocyte leukostasis in the retinas of RIRI mice peaking on the first day, followed by their extravasation on the second day post-injury. Concomitantly, microglial activation and proliferation begin as early as 2 hours post-ischemia and continue for a week [8, 35].In addition to these cellular changes, lymphocyte infiltration, especially of CD4^+^ IFN-γ-producing helper T cells (Th1), is evident and appears to exacerbate RGC damage. This retina infiltration starts on the second day post-injury, peaking on the seventh day for lymphocytes and two weeks for CD4^+^ T cells [8]. Interestingly, these cellular alterations correlate with a phase of inner retinal thinning, primarily due to apoptosis, that continues for approximately two weeks post-RIRI [8]. These cellular changes elucidate a sustained retinal immune response, including microglia activation and macrophage and lymphocyte infiltration. This immune response peaks on the seventh day post-injury and is followed by a period of resolution over the next three weeks [8]. The alternations in immune cell activity and the repair process are inextricably linked. Thus, delineating the temporal and spatial changes of these immune cells and understanding their specific functions may offer novel targets for RIRI intervention.

## 7. Future basic research directions

As we venture into the future of research on RIRI, a holistic and integrated approach is imperative. The exploration of this complex condition necessitates a convergence of multiple research paths, each interweaving with the others to form a comprehensive understanding.

At the forefront of this endeavor is the need to delve into the nuanced interactions between microglia and Müller cells [201]. These resident immune cells play a critical role in the inflammatory response and neuroprotection during RIRI. Understanding the mechanisms that dictate their interaction will be vital in identifying novel therapeutic targets to modulate these relationships beneficially.

Simultaneously, the dual role of astrocytes in RIRI demands attention [202, 203]. Research should aim to unravel the molecular triggers that influence astrocyte behavior, transitioning them between protective and harmful states. This knowledge is crucial for leveraging their neuroprotective potential while mitigating any detrimental effects.

Furthermore, the roles of peripheral immune cells such as T cells, neutrophils, and monocyte-derived macrophages in the pathophysiology of RIRI require deeper exploration. Studies focusing on their recruitment, specific contributions, and interactions with resident retinal cells could illuminate new paths for targeted immunomodulatory therapies [41].

An equally significant area of focus is the function of inflammasomes in RIRI. Investigating the triggers of inflammasome activation and the subsequent downstream effects could reveal novel intervention points, offering new therapeutic strategies [120, 121].

Parallel to these cellular and molecular studies, the complex network of cytokines and signaling pathways integral to RIRI represents a fertile ground for research. Understanding how these molecular cascades orchestrate the immune response and contribute to tissue damage is crucial for the development of therapies targeting critical molecules in these pathways.

The advancement of therapeutic agent development is another critical direction. This includes exploring new compounds that can effectively modulate microglia activation, astrocyte response, and peripheral immune cell infiltration [75].

Conducting comprehensive long-term studies to track the progression of RIRI will provide invaluable insights into its natural history and the evolution of immune responses. Such knowledge is essential for identifying optimal therapeutic windows and understanding the long-term impacts of RIRI.

Gene therapy and molecular interventions targeting specific genes or pathways implicated in RIRI present a promising research avenue. Exploring strategies to upregulate protective genes or downregulate deleterious ones could offer new therapeutic possibilities.

Advancements in imaging and diagnostic techniques are also crucial [204]. Enhancing these technologies will not only deepen our understanding of RIRI but also aid in its early detection and monitoring. Such advancements are vital for assessing the efficacy of new treatments and for tailoring patient-specific therapeutic strategies.

Lastly, the integration of multi-omics approaches (genomics, proteomics, metabolomics) in RIRI research will enable a more comprehensive understanding of the disease at the molecular level [30, 196, 205-207]. This approach is anticipated to uncover novel biomarkers and therapeutic targets, paving the way for personalized medicine in RIRI management.

In summary, the future research landscape of RIRI is a tapestry of interrelated studies, each contributing to a more profound understanding of the condition. This comprehensive approach aims not only to deepen our understanding of RIRI but also to translate these insights into more effective and targeted treatments for this debilitating condition.

## 8. Future translating retinal ischemia research: from laboratory insights to clinical applications

Translating the current understanding of RIRI into clinical practice necessitates a seamless integration of diverse strategies, focusing on both diagnostic advancements and therapeutic innovations.

Central to this endeavor is the identification and utilization of biomarkers within the unique immune microenvironment following retinal ischemia [208, 209]. The intricate interplay of immune cells, neurons, cytokines, and signaling pathways in RIRI subtly influences the outcome of ischemic injury. By analyzing inflammatory factors and antibodies resultant from retinal damage, gleaned from collected intraocular fluids like aqueous humor, vitreous fluid, and subretinal fluid, researchers can uncover insights into chronic and subclinical microalterations [210]. Proteomics research methods will play a crucial role in isolating and identifying proteins from these fluids. Differentially expressed proteins, once screened and verified for their expression levels using sensitive detection platforms, will lead to the discovery of biomarkers with strong diagnostic efficacy [211]. The integration of these biomarkers with multi-omics and artificial intelligence methods will significantly enhance their applicability in diagnosis [211, 212], treatment efficacy evaluation, and prognosis assessment of retinal ischemic diseases.

In parallel, the combination of these intraocular fluid biomarkers with advanced imaging techniques and comprehensive clinical data is set to revolutionize the diagnostic process [204]. This multifaceted approach not only enables more precise diagnoses but also facilitates the customization of treatments to individual patients’ needs, alongside the accurate monitoring of treatment efficacy and prognosis assessment.

The transition from research to clinical application also involves the development and rigorous testing of new therapeutic agents. The promising efficacy demonstrated by potential therapeutic targets such as TLR4/NF-κB/NLRP3 pathway inhibitors and agents that promote the M2 macrophage phenotype in animal models lays a solid foundation for clinical translation. The development of immune agents targeting these key factors, potentially administered through intravitreal injections, is poised to improve the prognosis of ischemic retinal damage. The gradual verification of the safety and effectiveness of these treatments, achieved through meticulously designed clinical trials based on preclinical research results, will be a critical step in bringing these advances to patients.

Bridging the gap between preclinical and clinical research is a critical aspect of this translation. This involves not only applying findings from animal models to human patients but also adapting these findings to the unique complexities of human physiology and disease progression. Collaborative efforts between researchers, clinicians, and pharmaceutical companies are essential to ensure a smooth transition from laboratory to clinic.

In summary, the journey from understanding RIRI at a molecular level to applying this knowledge in clinical settings is a multifaceted process, requiring a convergence of biomarker research, advanced imaging, therapeutic development, and a bridge between preclinical and clinical research. This comprehensive approach promises to significantly enhance the management of patients suffering from retinal ischemic diseases.

## 9. Summary

To sum up, the immune system responds to RIRI, with different immune cells playing specific roles in damaging, protecting, and repairing the retina. In this assessment, we've probed the current knowledge and obstacles in comprehending the role of immune cells in RIRI, identifying key research avenues for future explorations and clinical translation. Research has underscored the participation of multiple immune cell groups, such as microglia, invading monocytes/macrophages, T cells, and neutrophils in RIRI. Nonetheless, the distinct roles and interconnections of these cells within the retinal context remain somewhat ambiguous. Upcoming studies should leverage sophisticated methods to demystify the distinct roles and shifts of immune cell categories during RIRI. Additionally, examining the interactions between resident immune cells and peripheral immune cells is essential for a thorough comprehension of RIRI. The molecular communication routes and interaction strategies among microglia, invading monocytes/macrophages, and T cells should be clarified to ascertain their collective influence on retinal inflammation and neural injury.

Inflammasomes have been spotlighted as vital contributors in RIRI, but the exact molecular pathways driving their engagement and subsequent effects on neural injury warrant more profound study. Understanding the inducers and signaling activities tied to inflammasome triggering will offer crucial insights for specialized therapeutic approaches. In essence, creating specialized therapeutic methodologies remains an imperative avenue for future RIRI studies. Through the adjustment of immune cell response and orientation, curbing inflammasome triggering, and directing the neuroinflammatory sequence, we might be able to reduce retinal injury and sustain visual capability.

To wrap up, we emphasize the significance of investigating the interactions between resident immune cells and peripheral immune cells in RIRI. By addressing the existing challenges and adopting the proposed areas of investigation, we can delve deeper into the fundamental processes and develop pioneering methods for preventing and managing retinal ischemic ailments. The profound insights derived from these pursuits offer substantial potential for enhancing clinical results and sustaining visual capabilities in individuals impacted by RIRI.
